# Analysis of nontuberculous mycobacteria laboratory detection data from a designated hospital in Henan Province, China

**DOI:** 10.1186/s12866-026-04736-y

**Published:** 2026-01-19

**Authors:** Jiao Tan, Honghua Zhang, Yachun Wang, Xue Han, Chen Chen, Nan Zang, Xu Hu, Jingcai Gao, Wei Wang

**Affiliations:** 1 Department of Medical Laboratory, Henan Provincial Chest Hospital, Zhengzhou University, Zhengzhou, Henan, 450000 China; 2 Henan Provincial Key Laboratory of Tuberculosis Diagnosis Medicine, Zhengzhou, Henan, 450000 China; 3 Henan Provincial Engineering Research Center for Detection and Treatment of Multidrug-Resistant Tuberculosis, Zhengzhou, Henan, 450000 China; 4 Henan Provincial Key Medical Discipline - Laboratory Diagnostics, Zhengzhou, Henan, 450000 China; 5 Department of Cardiac Intensive Care Unit, Henan Provincial Chest Hospital, Zhengzhou University, Zhengzhou, Henan, 450000 China; 6 Department of Internal Medicine-Tuberculosis, Henan Provincial Chest Hospital, Zhengzhou University, Zhengzhou, Henan, 450000 China; 7 Department of Research and Science, Henan Provincial Chest Hospital, Zhengzhou University, Zhengzhou, Henan, 450000 China

**Keywords:** Nontuberculous mycobacteria, Species identification, Mycobacterial culture, GeneXpert MTB/RIF, Mycobacterium tuberculosis

## Abstract

**Background:**

Nontuberculous mycobacteria (NTM) infections are emerging globally, yet epidemiological data from central China remain limited. This study analyzed mycobacterial culture and identification data from Henan Provincial Chest Hospital (2020–2024) to assess NTM detection patterns and species distribution.

**Methods:**

This retrospective laboratory surveillance study analyzed mycobacterial culture and species identification data from Henan Provincial Chest Hospital (January 2020–December 2024). Cultures were performed using the MGIT system with MPB64 antigen detection for MTBC and PNB growth for presumptive NTM identification. Species identification employed DNA microarray targeting the 16–23 S rRNA internal transcribed spacer region. Clinical data (symptoms, radiology, comorbidities), repeated cultures per ATS guidelines, and treatment outcomes were not available. GeneXpert MTB/RIF-negative cases with cycle threshold less than 42 from 2022 to 2024 were analyzed retrospectively.

**Results:**

Among 6,939 culture-positive specimens, 962 (13.86%) were identified as NTM, with annual isolation rates increasing from 10.76% to 16.37%. Of these 962 NTM cases, 532 isolates (55.3%) underwent DNA microarray species identification, revealing *M. intracellulare* as predominant (79.51%, 423/532), followed by *M. abscessus* complex (10.53%, 56/532), *M. kansasii* (4.32%, 23/532), *M. avium* (3.01%, 16/532), and *M. fortuitum* (1.69%, 9/532). Among 532 NTM isolates undergoing species identification, males (59.77%) and elderly patients (> 60 years) predominated. In a subgroup of 230 patients with GeneXpert MTB/RIF-negative results but cycle threshold (Ct) values < 42, 101 were identified as NTM-positive. Notably, 94.25% of these cases were attributed to the *Mycobacterium avium* complex (MAC), with 93 showing low Ct for probe A and 7 for probe C.

**Conclusions:**

NTM isolation rates are rising in Henan Province, with *M. intracellulare* as the predominant species. GeneXpert-negative cases with low Ct values for probe A may benefit from supplementary acid-fast staining and species identification to enhance early NTM detection, findings represent laboratory detection patterns from single specimens and cannot distinguish colonization from clinically significant disease per ATS/IDSA diagnostic criteria.

## Introduction

Nontuberculous mycobacteria (NTM) refer to a diverse group of *mycobacterial* species that do not belong to the *Mycobacterium tuberculosis* complex (MTBC)—which includes *Mycobacterium tuberculosis* (MTB), *Mycobacterium bovis*, and others—or to *Mycobacterium leprae* and *Mycobacterium lepromatosis* [1]. To date, more than 190 validly published NTM species and subspecies have been identified according to current taxonomic databases. The majority are environmental saprophytes found in water and soil, with only a limited number recognized as clinically significant opportunistic human pathogens [2, 3].

NTM are widely distributed across the globe, with regional variation in prevalence and clinical presentation. Epidemiological surveys over the past decades have shown a significant upward trend in the isolation rate of NTM in China, increasing from 4.3% in 1979 to 11.1% in 2000, and reaching 22.9% in 2010, indicating a rising incidence of NTM infections nationwide [4, 5]. NTM can affect virtually all organs and systems of the human body, primarily entering through the respiratory tract, gastrointestinal tract, and skin [6]. The pathogenesis and clinical manifestations of NTM infection closely resemble those of tuberculosis (TB), with similar systemic symptoms and local lesions, making differentiation between the two conditions clinically challenging [7]. However, unlike MTB, most NTM species exhibit intrinsic resistance to first-line anti-TB drugs, which complicates treatment strategies. Therefore, accurate and timely identification of NTM species is of critical importance in clinical practice [8, 9]. In this study, we analyzed data from the Henan Provincial Chest Hospital, including mycobacterial culture results, species identification by DNA microarray, and GeneXpert MTB/RIF testing, to evaluate the prevalence of NTM infections, species distribution, demographic characteristics of affected individuals, and the clinical relevance of GeneXpert-negative cases in diagnosing NTM infections. The aim was to provide a reference for the improvement of diagnostic techniques and epidemiological understanding of NTM infections at this institution.

This retrospective laboratory-based study analyzed mycobacterial culture and species identification data from Henan Province (2020–2024) to characterize NTM detection patterns and species distribution, supporting regional surveillance efforts.

## Materials and methods

### Sample collection

Clinical specimens were collected between January 2020 and December 2024 as part of routine diagnostic workup for patients with suspected mycobacterial infections at Henan Provincial Chest Hospital. The majority of samples were sputum, obtained from patients in the outpatient respiratory clinics, inpatient pulmonary wards, and the tuberculosis department. A minority of specimens consisted of bronchoalveolar lavage fluid (BALF) and other respiratory tract samples.To avoid duplication, only the first culture-positive isolate per patient during 2020–2024 was included. Among 962 culture-positive NTM cases, 532 isolates (55.3%) underwent DNA microarray species identification. Repeated cultures were not systematically tracked; therefore we cannot assess how many patients had two or more positive cultures, the time intervals between cultures, or whether repeat isolates showed the same or different species. This prevents assessment of the ATS/IDSA criterion requiring at least two positive cultures for NTM pulmonary disease diagnosis. This study was approved by the Ethics Committee of Henan Provincial Chest Hospital. As the study involved anonymized, retrospective data, the requirement for informed consent was waived.

### Reagents and materials

The following reagents and diagnostic kits were used in the study: MGIT culture tubes (BD, USA), MPB64 antigen detection kit (Hangzhou GENESIS Biodetection & Biocontrol Co., Ltd), *p*-Nitrobenzoic acid (PNB) culture tubes (Zhuhai BESBIO, China), Mycobacterial species identification chip kit (CapitalBio, Chengdu, China), GeneXpert MTB/RIF assay kit (Cepheid, Shanghai, China), Acid-fast staining reagents using cold staining method (Zhuhai BESBIO, China).

### Procedures

#### Mycobacterial culture

Mycobacterial cultures were performed in accordance with standard protocols for liquid culture of mycobacterial specimens. Clinical specimens from patients with suspected mycobacterial infections were collected and processed using the NALC-NaOH digestion–centrifugation method. Cultures were incubated in MGIT tubes and monitored using the BD BACTEC MGIT 960 automated mycobacterial detection system. Upon a positive signal, acid-fast staining was performed. If the acid-fast stain was positive, 2–3 drops of culture fluid were added to the MPB64 antigen detection kit. A positive MPB64 result was interpreted as *Mycobacterium tuberculosis* complex (MTBC) culture positivity. If negative, the culture was subcultured into PNB-containing tubes. Growth in PNB medium combined with positive acid-fast staining was interpreted as indicative of NTM.

DNA extraction.

Genomic DNA was extracted from MGIT culture-positive isolates using the boiling method. Upon positive signal from the BACTEC MGIT 960 system and confirmation of acid-fast positivity, 2–3 drops of culture suspension were transferred to a 1.5 mL microcentrifuge tube containing 200 µL sterile distilled water. The suspension was vortexed briefly, heated at 100 °C for 10 min in a dry bath incubator, then immediately cooled on ice for 2 min. The mixture was centrifuged at 12,000 rpm (approximately 13,400 × g) for 5 min at room temperature. The supernatant (~ 150–180 µL) containing genomic DNA was carefully transferred to a new sterile tube and used immediately for microarray analysis or stored at − 20 °C for up to one week.

#### Species identification by DNA microarray

Species identification was performed using the CapitalBio Mycobacterium Detection Array (CapitalBio Corporation, Chengdu, China), which utilizes 16–23 S rRNA internal transcribed spacer (ITS) region analysis.

1. PCR amplification: The ITS region was amplified using fluorescently labeled primers provided in the kit. PCR reactions (25 µL total volume) contained 5 µL extracted DNA template, 12.5 µL PCR master mix, 1 µL each of forward and reverse primers, and 5.5 µL nuclease-free water. Thermal cycling conditions: initial denaturation 95 °C for 5 min; 35 cycles of 95 °C for 30 s, 60 °C for 30 s, 72 °C for 45 s; final extension 72 °C for 7 min.
2. Hybridization: PCR products (5 µL) were mixed with 20 µL hybridization buffer, denatured at 95 °C for 5 min, and immediately applied to pre-warmed microarray chips (50 °C). Hybridization proceeded at 50 °C for 60 min in a humid chamber.
3. Washing and detection: Chips were washed twice with washing buffer at 50 °C (5 min each) to remove non-specific binding, dried by centrifugation, and scanned using the CapitalBio LuxScan 10 K microarray scanner.
4. Data analysis: Fluorescent signal patterns were analyzed using manufacturer’s software. Species identification was achieved by comparing hybridization patterns to a database containing > 100 mycobacterial species-specific probe signatures.
5. Quality control: Each run included positive controls (M. tuberculosis H37Rv DNA) and negative controls (no-template PCR). Only results with clear hybridization signals and no cross-reactivity were considered valid.

### GeneXpert MTB/RIF testing

GeneXpert MTB/RIF-negative cases from 2022 to 2024 were retrospectively analyzed. Cases with negative results but cycle threshold (CT) values less than 42 were selected for further clinical and microbiological review.

### Statistical analysis

Data on species identification and patient demographics were collected and organized using Microsoft Excel 2019 (Microsoft Corporation, Redmond, Washington, USA). Statistical analyses, including calculation of percentages and descriptive statistics, were conducted using IBM SPSS Statistics version 26.0 (IBM Corporation, Armonk, New York, USA).

## Results

### Mycobacterial culture findings

#### Distribution of culture-positive strains

A total of 6,939 mycobacterial isolates were obtained from culture-positive clinical specimens between 2020 and 2024. Among these, 5,977 isolates were identified as MTBC, and 962 as NTM, corresponding to an overall NTM isolation rate of 13.86% (962/6,939) (Table 1). Detailed clinical metadata such as smoking history, comorbidities (e.g., immunosuppression, structural lung disease), or treatment outcomes were not available due to the retrospective and laboratory-based nature of the dataset. All specimens analyzed were derived from respiratory sources, primarily sputum and BALF. No extrapulmonary samples were submitted to the laboratory during the study period.

**Table 1:** Isolation rates of NTM from mycobacterial cultures

Species group	Number of cases	Proportion (%)
MTBC	5977	86.14%
NTM	962	13.86%
Total	6939	100%

*MTBC *Mycobacterium tuberculosis complex, *NTM* Nontuberculous mycobacteria

### Demographic characteristics of patients with NTM-positive cultures

**Fig. 1:**
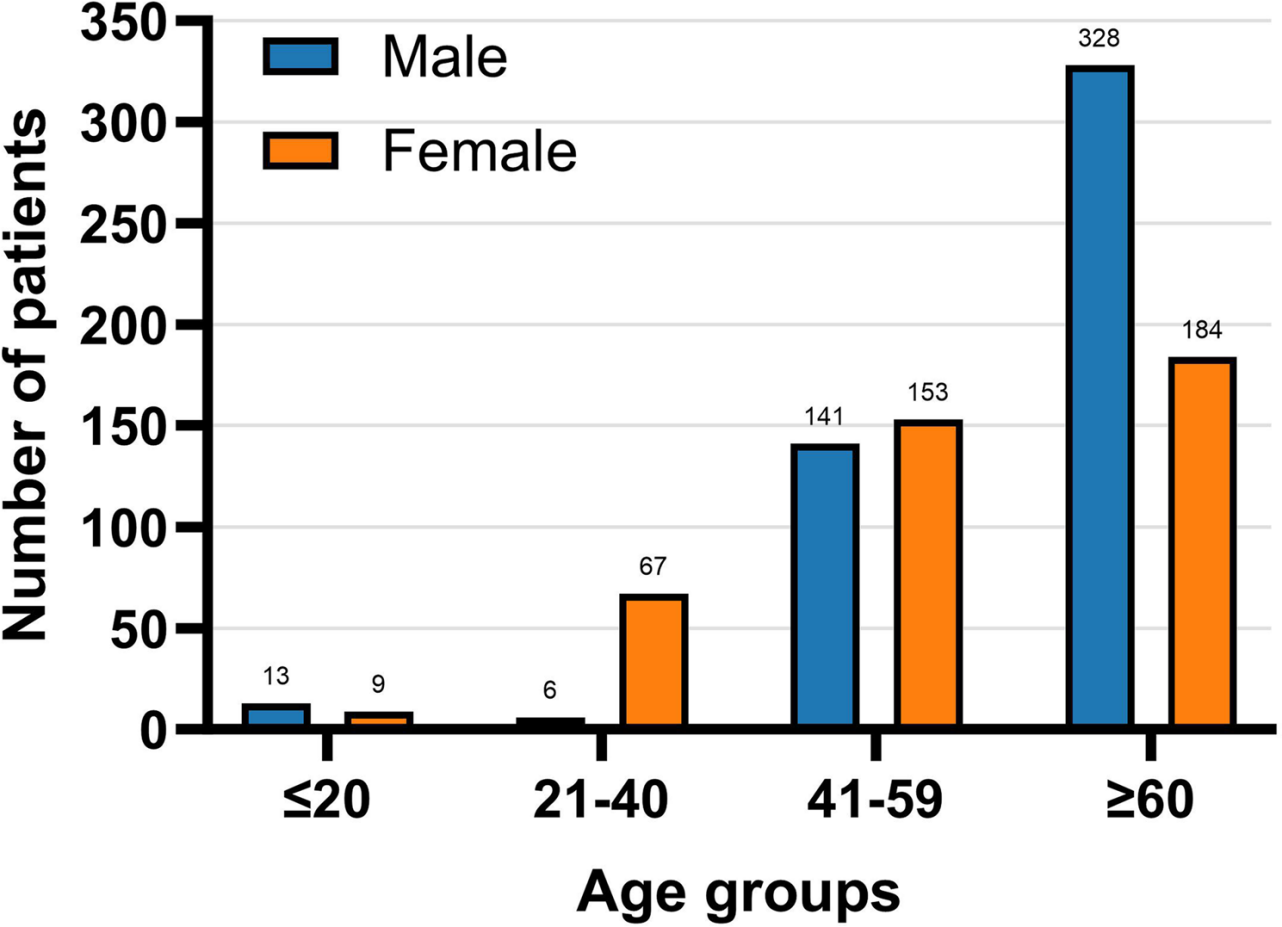
Age and sex distribution of patients with NTM-positive cultures.Distribution of 962 culture-confirmed NTM patients by age group and sex. The highest proportion of cases occurred in patients aged over 60 years, particularly among males. NTM, nontuberculous mycobacteria

Among the 962 patients with culture-confirmed NTM infection, 545 (56.65%) were male and 417 (43.35%) were female. Patients were stratified into four age groups: <20 years, 21–40 years, 41–60 years, and > 60 years. The highest number of cases occurred in patients aged over 60 years. In this age group, the proportion of male patients was notably higher at 60.18% (328/545) compared to 44.12% (184/417) in females. The age and sex distribution of NTM cases is further illustrated in Fig. 1.

### Annual statistics of NTM Isolation, 2020–2024

**Fig. 2:**
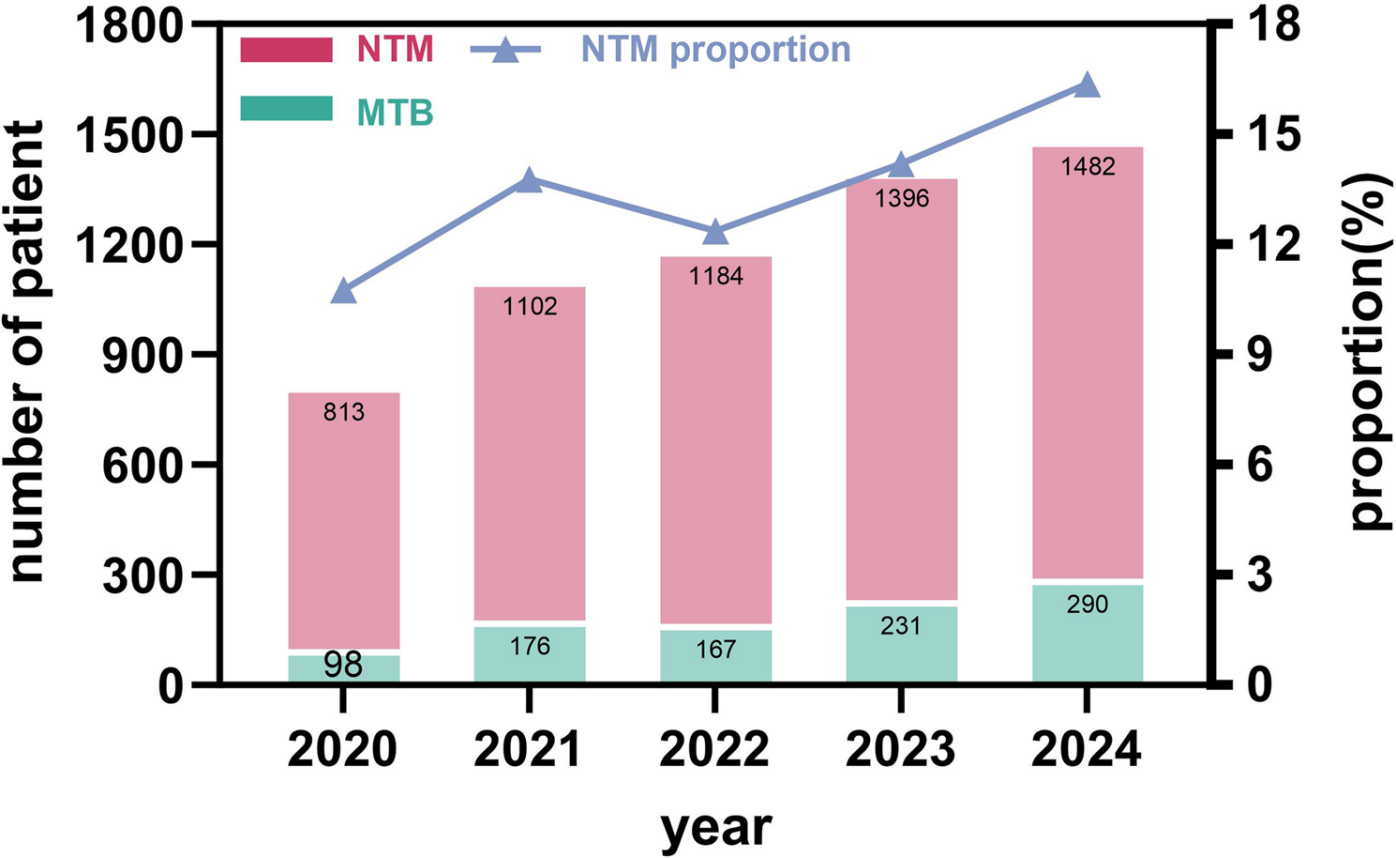
Annual trend of NTM isolation rate at Henan Chest Hospital from 2020 to 2024. Annual number of MTBC (teal) and NTM (pink) isolates with NTM proportion (line graph with triangles) among all mycobacterial culture-positive specimens from 2020 to 2024. *Note: Rates represent laboratory isolation from single specimens without clinical correlation; not clinically confirmed disease prevalence. NTM, nontuberculous mycobacteria; MTBC, *Mycobacterium tuberculosis* complex

From 2020 to 2024, the isolation rates of NTM at Henan Chest Hospital showed a generally increasing trend. In 2020, a total of 911 mycobacterial isolates were obtained, among which 813 were identified as MTBC, and 98 were NTM, resulting in an NTM isolation rate of 10.76% (98/911). In 2021, 1102 MTBC and 176 NTM strains were isolated, with an NTM isolation rate of 13.77% (176/1278). The NTM isolation rate slightly decreased in 2022 to 12.36% (167/1351), followed by an increase in 2023 to 14.20% (231/1627), and reached 16.37% (290/1772) in 2024. The annual trend of NTM isolation is illustrated in Fig. 2.

### Species identification of isolates

#### NTM detection rate

From 2020 to 2024, a total of 2,642 positive isolates underwent species identification. This included 516 (98.5%) of 524 culture-confirmed NTM cases, excluding mixed infections and repeat isolates. Among these, 2,110 were identified as *M. tuberculosis*, and 16 were mixed infections involving both MTBC and NTM. A total of 516 isolates were confirmed as NTM, yielding a cumulative NTM detection rate of 20.13% (532/2642). Drug susceptibility testing (DST) was not performed for NTM isolates during the study period, and corresponding resistance profiles were therefore not available for analysis. The detailed annual data are presented in Table 2.

**Table 2:** NTM detection rate based on species identification, 2020–2024

Species identified	Number of cases	Proportion (%)
MTBC	2110	79.86%
NTM species:		
*Mycobacterium intracellulare*	423	79.51
*Mycobacterium abscessus complex*	56	10.53
*Mycobacterium kansasii*	23	4.32
*Mycobacterium avium*	16	3.01
*Mycobacterium fortuitum*	9	1.69
*Mycobacterium smegmatis*	2	0.38
*Mycobacterium gordonae*	1	0.19
*Mycobacterium szulgai*	1	0.19
*Mycobacterium aurum*	1	0.19
NTM subtotal	532	20.13
Total	2642	100

*MTBC *Mycobacterium tuberculosis complex, *NTM *Nontuberculous mycobacteria

*Note: NTM subtotal includes 516 pure NTM isolates and 16 mixed MTBC/NTM infections detected in single specimens

### Demographic distribution of patients with NTM-positive isolates

Among the 532 patients with species-identified NTM, 318 (59.77%) were male and 214 (40.23%) were female. The demographic pattern was consistent with the full cohort of 962 culture-positive NTM patients (Fig. 1), showing male predominance and highest case numbers in the over 60 years age group, with no selection bias between culture-based and molecular identification methods.

### Species distribution of NTM isolates (2020–2024)

Species identification of the 532 NTM isolates collected from 2020 to 2024 revealed the predominance of *Mycobacterium intracellulare* (Int), which accounted for 79.51% (423/532) of cases. Note that all *M. intracellulare* isolates reported may include *M. chimaera* and *M. yongonense*, requiring sequencing-based methods for definitive subspecies identification. Other species included: *Mycobacterium abscessus* complex (CA) in 56 cases (10.53%), *Mycobacterium kansasii* (Kan) in 23 cases (4.32%), *Mycobacterium avium* (Avi) in 16 cases (3.01%), *Mycobacterium fortuitum* (For) in 9 cases (1.69%), and *Mycobacterium smegmatis* (Sme) in 2 cases (0.38%). Rare isolates included *Mycobacterium gordonae* (Gor), *Mycobacterium szulgai* (SM), and *Mycobacterium aurum* (Aur), each detected in a single case (0.19%). The distribution of NTM species is shown in Table 3.

**Table 3:** Annual distribution of NTM species identified by DNA microarray, 2020–2024

Species	2020	2021	2022	2023	2024	Total	%
*M. intracellulare*	18	91	75	107	132	423	79.51
*M. abscessus* complex	2	12	11	9	22	56	10.53
*M. kansasii*	2	6	2	6	7	23	4.32
*M. avium*	0	5	4	2	5	16	3.01
*M. fortuitum*	0	2	2	1	4	9	1.69
Other NTM specie	0	1	0	1	3	5	0.94
NTM subtotal	22	117	94	126	173	532	100

M *Mycobacterium*, *NTM *Nontuberculous mycobacteria. Other NTM species: *M. smegmatis* (*n* = 2), *M. gordonae* (*n* = 1), *M. szulgai* (*n* = 1), *M. aurum* (*n* = 1). *M. intracellulare* predominated consistently across all years, ranging from 76.3% to 84.9% annually

### GeneXpert-negative data analysis

#### Retrospective analysis of GeneXpert MTB/RIF-negative cases with Ct values < 42

**Fig. 3:**
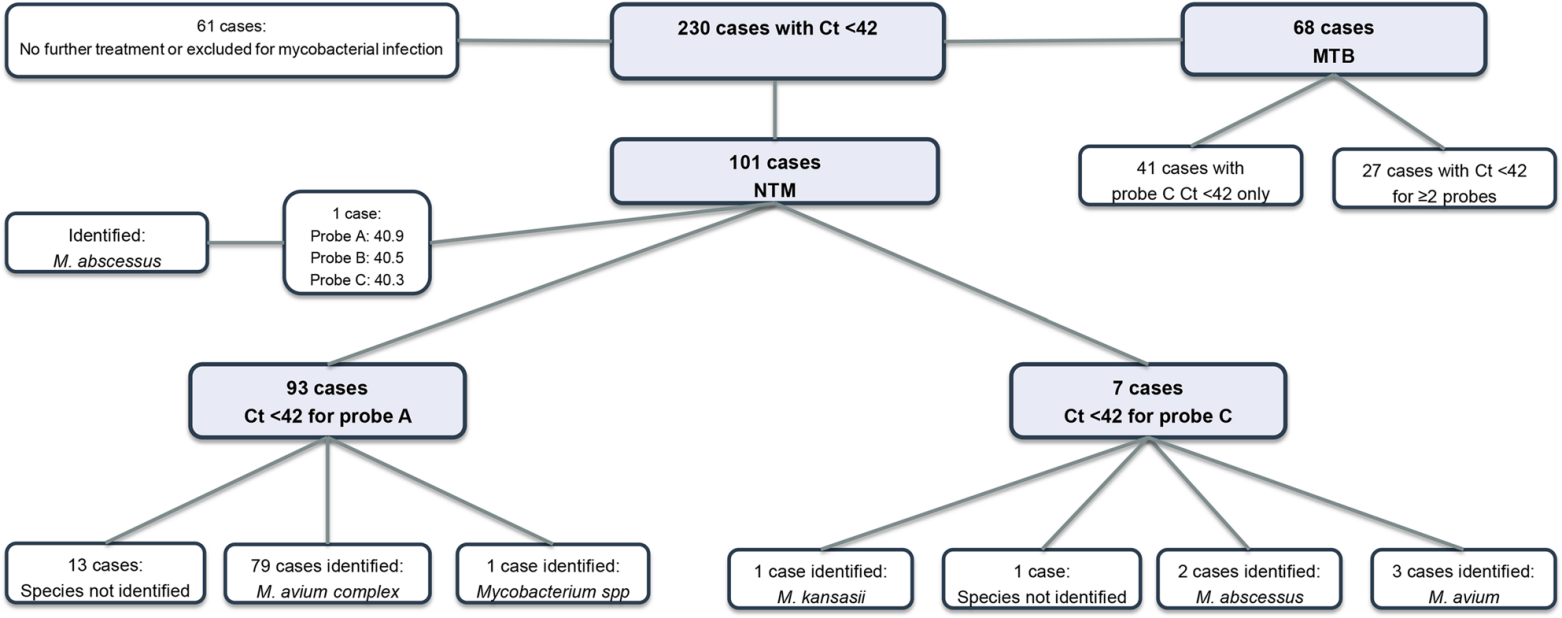
Distribution of GeneXpert probe A and C Ct values in culture-confirmed NTM and MTB cases. Patients with Ct < 42 for probe A or C were more likely to have NTM, such as MAC, despite being GeneXpert-negative for MTB. Ct, cycle threshold; NTM, nontuberculous mycobacteria; MTB, *Mycobacterium tuberculosis*; MAC, *Mycobacterium avium* complex

From 2022 to 2024, 230 patients with negative GeneXpert MTB/RIF results but Ct values < 42 were selected for further analysis. The Ct < 42 cutoff was determined based on internal retrospective review of laboratory patterns and not derived from standardized diagnostic guidelines. This threshold was used to explore potential diagnostic utility and is not intended as a validated clinical criterion. A total of 230 such cases were identified. Among them, 61 patients were either lost to follow-up, did not undergo further diagnostic evaluation, or were ruled out for mycobacterial infection. Of the remaining 169 cases, 68 were ultimately diagnosed with tuberculosis (MTB), while 101 were confirmed to have NTM infections. Of the 101 NTM-positive isolates, 93 had CT value exhibited a Ct < 42 for probe A only, with Ct values ranging from 29.5 to 40.1. Seven isolates had Ct < 42 for probe C only (range: 38.7–40.2), and one isolate exhibited Ct values < 42 for probes A, B, and C (40.9, 40.5, and 40.3, respectively). Of these 101 NTM-positive isolates, 87 underwent species identification. The majority (82/87, 94.25%) were identified as *Mycobacterium avium-intracellulare* complex (MAC). This proportion was notably higher than the overall cohort MAC rate (82.52%, 439/532), suggesting enrichment in GeneXpert-negative samples with low Ct values. The DNA microarray platform can differentiate *M. avium* from *M. intracellulare*; however, technical factors sometimes result in identification only to complex level. Given *M. intracellulare* predominance in our overall cohort (ratio 26:1 compared to *M. avium*), we infer that the majority of these 82 MAC cases likely represent *M. intracellulare* infections, though definitive confirmation requires sequencing-based methods such as hsp65 or rpoB gene analysis. Among these 82 MAC cases, 79 (96.3%) had probe A Ct < 42 only, while 3 (3.7%) had probe C Ct < 42 only.” The predominance of probe A positivity further supports this inference, as *M. intracellulare* typically triggers probe A in GeneXpert assays. Four isolates (4.60%) were identified as *Mycobacterium abscessus* complex, of which two had probe C Ct < 42, one had a probe A Ct of 40.1, and one had Ct values < 42 for all three probes (A, B, and C). One isolate was identified as *Mycobacterium kansasii*, with a probe C Ct value of 39.9. In the 68 MTB-positive isolates, 41 (60.29%) had Ct < 42 for probe C only, while 27 (39.71%) had Ct values < 42 for two or more probes. Due to lack of clinical data (e.g., radiology, symptoms), we were unable to assess whether GeneXpert-negative but Ct < 42 cases met ATS/IDSA diagnostic criteria for NTM pulmonary disease. The distribution of probe-specific Ct values among GeneXpert-negative but ultimately diagnosed cases is shown in Fig. 3.

## Discussion

NTM are widely distributed in natural environments [10] and are recognized as opportunistic pathogens with increasing clinical significance. In recent years, the isolation rate of NTM in China has shown a fluctuating upward trend [11], posing a growing public health challenge and raising concerns regarding its threat to human health. Globally, the burden of NTM disease has drawn increasing attention from researchers; however, due to the lack of mandatory reporting in most countries, accurate epidemiological data on NTM infections remain limited [12]. As clinical data were unavailable, we did not apply diagnostic criteria for NTM pulmonary disease, such as those proposed by the ATS/IDSA in 2007 or 2020 [13]. Our analysis was limited to microbiologically confirmed NTM isolates rather than confirmed NTM-PD cases.

According to the literature, population aging is considered a major contributing factor to the rising NTM isolation rate [3]. In this study, conducted at the largest designated tuberculosis hospital in Henan Province, the overall NTM isolation rate was 13.86%. The highest number of cases occurred in patients aged over 60 years, with a higher prevalence in males than females. A clear age-related trend was observed, with the NTM isolation rate increasing significantly with age, reflecting a prominent aging pattern among affected individuals. The species identification of NTM using DNA microarray demonstrated a higher detection rate compared to traditional mycobacterial culture. This discrepancy is likely attributable to methodological differences. In clinical practice, DNA microarray is more frequently employed for patients suspected of NTM infection, whereas other molecular techniques such as TB-DNA and GeneXpert MTB/RIF are preferred for the identification of MTBC. Despite these methodological differences, the demographic distribution (in terms of age and sex) revealed similar patterns across both methods, with males being more frequently affected than females, and elderly males (> 60 years) constituting the majority. These findings are consistent with the results reported by Verma A.K. et al. [14], and further support the hypothesis that underlying comorbidities and immunosuppression may serve as important risk factors for NTM disease. Consistent with the studies by Khare R [15]. and Shen Y [16]. our investigation also revealed limitations in culture-based methods, particularly the inability to distinguish co-infection with NTM and MTB due to overlapping growth characteristics. In contrast, species identification using DNA microarray enables the simultaneous detection of both NTM and MTB in a single specimen. These findings underscore the importance of raising clinical awareness: in cases where patients respond poorly to standard anti-tuberculosis or anti-NTM therapy, the possibility of mixed infections should be considered and investigated accordingly.

Over the five-year study period, we observed a gradual increase in the NTM isolation rate, rising from 10.76% in 2020 to 16.37% in 2024. This trend may be attributed to several converging factors. First, demographic changes, particularly the increasing proportion of elderly individuals in China, may contribute to rising susceptibility to opportunistic infections such as NTM [3, 17]. Second, environmental influences, including air pollution, urbanization, and climate variation, may facilitate human exposure to environmental mycobacteria [18]. Third, diagnostic practices have evolved over time, with increased use of molecular platforms such as GeneXpert and DNA microarrays likely improving detection sensitivity. Together, these factors may explain the temporal rise in NTM detection in our center.

While our findings indicate a predominance of elderly male patients with NTM isolation, studies from neighboring countries such as South Korea, Japan, and India have reported a notable increase in NTM pulmonary disease among younger adults, particularly women aged 30–50 years [19, 20]. This discrepancy may reflect regional differences in environmental exposures, genetic predisposition, and underlying comorbidities such as bronchiectasis and prior tuberculosis. For example, in Korea and Japan, *M. avium* infection is often observed in non-smoking middle-aged women with nodular bronchiectatic disease—a pattern less common in our cohort. These comparisons underscore the importance of region-specific surveillance and risk factor analysis.

The predominance of *M. intracellulare* and *M. abscessus* in our isolates highlights the need for species-specific clinical vigilance. While MAC infections are generally indolent and may respond to macrolide-based regimens, *M. abscessus* is associated with high levels of intrinsic and inducible macrolide resistance [21]. Subspecies-level identification is important for both *M. abscessus* and *M. intracellulare* complexes, as different subspecies exhibit distinct drug resistance profiles and clinical behaviors. The specific implications of this limitation are discussed in the Limitations Sect [22].

With the advancement of diagnostic technologies, accurate differentiation of NTM species has become increasingly feasible [23]. However, as NTM are environmental opportunistic pathogens predominantly found in water and soil, their isolation rates and species distribution vary significantly across different geographical regions. These variations are influenced by multiple factors, including geography, climate, environmental conditions, economic development, cultural practices, and healthcare infrastructure [24]. Globally, the most commonly reported NTM species include the *Mycobacterium avium* complex (MAC), *Mycobacterium abscessus* complex, *Mycobacterium kansasii*, and *Mycobacterium fortuitum* [3]. Even within China, interprovincial differences in NTM species distribution have been widely documented [17, 24–26]. In this study, the five most frequently isolated NTM species were: *Mycobacterium intracellulare*, *Mycobacterium abscessus* complex, *Mycobacterium kansasii*, *Mycobacterium avium*, and *Mycobacterium fortuitum*. The majority of cases were from Henan Province, which lies in a transitional zone between a temperate monsoon climate and a subtropical monsoon climate. This region features distinct seasonal changes, uneven precipitation, and well-developed transportation infrastructure—all of which may influence the environmental distribution and human exposure to NTM.

GeneXpert MTB/RIF, a molecular diagnostic tool based on real-time fluorescence PCR technology [27], is highly specific for the detection of MTBC. Several studies have also explored its potential utility in NTM diagnosis [28–31]. In the present study, we analyzed GeneXpert-negative samples with Ct values < 42. Notably, in cases where only probe A showed a Ct value < 42, subsequent acid-fast staining and species identification substantially increased the NTM detection rate. Among GeneXpert-negative cases with Ct < 42, species identification revealed that 79 patients were infected with MAC, accounting for 94.25% of typed isolates in this subgroup. This proportion was markedly higher than the MAC detection rate observed in routine DNA microarray-based typing across the full NTM cohort (82.52%, 439/532). These findings reaffirm that *M. intracellulare* remains the predominant NTM species in Henan Province. In summary, GeneXpert-negative results should not be overlooked in clinical practice—especially in cases where probe A has a Ct value < 42. However, it must be emphasized that the Ct < 42 threshold was derived from retrospective, internal observations rather than from validated clinical studies. Receiver operating characteristic (ROC) analysis or positive predictive value assessments were not feasible due to the lack of patient-level outcome data. Thus, while our findings suggest a potential correlation between low probe A Ct values and MAC positivity, prospective clinical validation is required before adopting such thresholds in practice. It is important to recognize that the GeneXpert MTB/RIF assay was specifically designed for detecting MTBC, not NTM. The use of probe-specific Ct thresholds in this study was based on retrospective observation and should not be construed as a validated diagnostic criterion for NTM infection. While the observed correlation between low probe A or C Ct values and MAC identification is intriguing, further prospective clinical validation is required before such thresholds can be applied in routine diagnosis.

Looking ahead, this study has several limitations that warrant further investigation. First, the exclusive focus on respiratory specimens reflects the institutional role of Henan Provincial Chest Hospital as a specialized chest hospital. Extrapulmonary NTM infections such as lymphadenitis, skin infections, and disseminated disease are typically managed at general hospitals. This referral pattern limits generalizability to extrapulmonary NTM disease. Second, the retrospective design and lack of clinical data—such as radiographic findings, symptoms, immune status, comorbidities, and treatment outcomes—limited our ability to distinguish between colonization and true NTM disease. Moreover, as only the first culture-positive isolate per patient was analyzed and repeated cultures were not systematically tracked, we cannot distinguish transient colonization from persistent infection or determine whether patients met the ATS/IDSA criterion requiring at least two positive cultures for definitive diagnosis. Future research should incorporate patient-level clinical information, repeated culture data, and apply standardized diagnostic frameworks, such as the ATS/IDSA 2020 criteria, to ensure clinical relevance. Third, due to technical limitations of the DNA microarray platform, subspecies differentiation within both the *M. abscessus* complex (subsp. *abscessus*, *massiliense*, and *bolletii*) and *M. intracellulare* complex (*M. chimaera*, *M. yongonense*) was not performed. For *M. abscessus* complex, this limitation is clinically significant as *M. massiliense* typically lacks functional erm(41) and responds better to clarithromycin than other subspecies. For *M. intracellulare* complex, the inability to identify *M. chimaera* is concerning as this subspecies is associated with disseminated infections and healthcare-associated outbreaks linked to heater-cooler units in cardiac surgery. Future research should incorporate high-resolution molecular methods such as hsp65 or rpoB gene sequencing, multi-locus sequence analysis (MLSA), or whole-genome sequencing (WGS) for definitive subspecies identification to guide individualized therapy and infection control measures. Fourth, our study did not include drug susceptibility testing (DST), which is critical for guiding therapy. Prospective, multicenter studies that integrate microbiological, clinical, and molecular data—including DST and subspecies identification—are essential to improve diagnostic accuracy, guide tailored treatment, and support public health surveillance. Finally, these findings highlight the need to establish practical diagnostic algorithms that incorporate molecular tools such as GeneXpert and DNA microarrays in resource-limited settings, enabling earlier detection and better outcomes for NTM-infected patients [6].

## Data Availability

The data that support the findings of this study are available from the corresponding author upon reasonable request.

## Abbreviations

NTM: Nontuberculous mycobacteria
MTBC: Mycobacterium tuberculosis complex
MTB: Mycobacterium tuberculosis
MAC: Mycobacterium avium complex
AFB: Acid-fast bacilli
Ct: Cycle threshold
PNB: p-Nitrobenzoic acid
MGIT: Mycobacteria Growth Indicator Tube
BALF: Bronchoalveolar lavage fluid
DST: Drug susceptibility testing
ATS/IDSA: American Thoracic Society/Infectious Diseases Society of America
